# Transformer-Based Deep-Learning Algorithm for Discriminating Demyelinating Diseases of the Central Nervous System With Neuroimaging

**DOI:** 10.3389/fimmu.2022.897959

**Published:** 2022-06-14

**Authors:** Chuxin Huang, Weidao Chen, Baiyun Liu, Ruize Yu, Xiqian Chen, Fei Tang, Jun Liu, Wei Lu

**Affiliations:** ^1^ Department of Radiology, The Second Xiangya Hospital of Central South University, Changsha, China; ^2^ Department of Neurology, The Second Xiangya Hospital of Central South University, Changsha, China; ^3^ Infervision Medical Technology Co., Ltd., Ocean International Center, Beijing, China; ^4^ Clinical Research Center for Medical Imaging in Hunan Province, Changsha, China

**Keywords:** deep learning, demyelinating disease, differential diagnosis, MRI, multiple sclerosis, myelin oligodendrocyte glycoprotein antibody-associated disease, neuromyelitis optica spectrum disorder, transformer

## Abstract

**Background:**

Differential diagnosis of demyelinating diseases of the central nervous system is a challenging task that is prone to errors and inconsistent reading, requiring expertise and additional examination approaches. Advancements in deep-learning-based image interpretations allow for prompt and automated analyses of conventional magnetic resonance imaging (MRI), which can be utilized in classifying multi-sequence MRI, and thus may help in subsequent treatment referral.

**Methods:**

Imaging and clinical data from 290 patients diagnosed with demyelinating diseases from August 2013 to October 2021 were included for analysis, including 67 patients with multiple sclerosis (MS), 162 patients with aquaporin 4 antibody-positive (AQP4+) neuromyelitis optica spectrum disorder (NMOSD), and 61 patients with myelin oligodendrocyte glycoprotein antibody-associated disease (MOGAD). Considering the heterogeneous nature of lesion size and distribution in demyelinating diseases, multi-modal MRI of brain and/or spinal cord were utilized to build the deep-learning model. This novel transformer-based deep-learning model architecture was designed to be versatile in handling with multiple image sequences (coronal T2-weighted and sagittal T2-fluid attenuation inversion recovery) and scanning locations (brain and spinal cord) for differentiating among MS, NMOSD, and MOGAD. Model performances were evaluated using the area under the receiver operating curve (AUC) and the confusion matrices measurements. The classification accuracy between the fusion model and the neuroradiological raters was also compared.

**Results:**

The fusion model that was trained with combined brain and spinal cord MRI achieved an overall improved performance, with the AUC of 0.933 (95%CI: 0.848, 0.991), 0.942 (95%CI: 0.879, 0.987) and 0.803 (95%CI: 0.629, 0.949) for MS, AQP4+ NMOSD, and MOGAD, respectively. This exceeded the performance using the brain or spinal cord MRI alone for the identification of the AQP4+ NMOSD (AUC of 0.940, brain only and 0.689, spinal cord only) and MOGAD (0.782, brain only and 0.714, spinal cord only). In the multi-category classification, the fusion model had an accuracy of 81.4%, which was significantly higher compared to rater 1 (64.4%, p=0.04<0.05) and comparable to rater 2 (74.6%, p=0.388).

**Conclusion:**

The proposed novel transformer-based model showed desirable performance in the differentiation of MS, AQP4+ NMOSD, and MOGAD on brain and spinal cord MRI, which is comparable to that of neuroradiologists. Our model is thus applicable for interpretating conventional MRI in the differential diagnosis of demyelinating diseases with overlapping lesions.

## 1 Introduction

Inflammatory demyelinating diseases of the central nervous system (CNS) are important causes of nontraumatic neurological disabilities (1, 2). Multiple sclerosis (MS), neuromyelitis optica spectrum disorder (NMOSD), and myelin oligodendrocyte glycoprotein antibody-associated disease (MOGAD) are major disease entities in this field (3, 4). Increasing evidence indicates that NMOSD is an independent disorder associated with the expression of anti-aquaporin-4 (AQP4) antibodies rather than a variant of MS (5, 6). With the discovery of antibodies targeting myelin oligodendrocyte glycoprotein (MOG) in AQP4 antibody-negative NMOSD, MOGAD is now recognized as a unique immunological entity that is distinct from both MS and NMOSD (4, 7).

Indeed, MS, NMOSD and MOGAD exhibit divergent pathogeneses, treatment options for relapse prevention, and prognoses (8), and their diagnosis is mainly based on combined results involving clinical findings, radiological manifestations, and cerebrospinal fluid and serological tests. In conventional magnetic resonance imaging (MRI), bilateral periventricular white matter and cortical lesions are often considered typical features of MS (9), whereas longitudinally extensive transverse myelitis and posterior long-segment optic nerve lesions are more specific to NMOSD (10). MOGAD is considered to exhibit intermediate MRI features between those of MS and NMOSD (11). However, the MRI manifestations in some cases are indistinguishable among these conditions. Owing to the presence of overlapping clinical and radiological findings among these disorders, differential diagnosis can be challenging.

Autoantibody tests normally confer high sensitivity, but are invasive and time-consuming to obtain results, and conversion to an antibody-negative status may occur during the disease course (12). An improper choice of treatment may lead to disease deterioration. For example, disease-modifying therapies such as interferon beta and dimethyl fumarate are recommended as the standard treatment for MS but may exacerbate NMOSD and increase relapse rates (13, 14). Immunosuppressive agents such as azathioprine and mycophenolate mofetil are the first-line therapies for NMOSD and MOGAD. Moreover, several emerging therapies have shown different efficacies in controlling disease recurrence in patients with AQP4+ NMOSD and MOGAD (15). Thus, to reduce the delay from disease onset to appropriate treatment, thereby improving clinical benefits, there is an urgent need to develop an effective non-invasive approach for a rapid and precise differential diagnosis.

Many researchers have explored imaging differences among the three diseases using multi-model MRI sequences, indicating that the classification of MR image features can be helpful in the diagnosis of these diseases. Duan et al. (16) compared brain structural alterations on MRI, and demonstrated cortical and subcortical atrophy without severe white matter rarefaction in MOGAD in comparison with MS and AQP4+ NMOSD, whereas diffusion MRI measurements showed lower fractional anisotropy and higher mean diffusivity in MS. Moreover, Banks et al. (17) retrospectively compared the involvement of the brainstem or cerebellar region in CNS inflammatory demyelination diseases, and revealed that diffuse middle cerebellar peduncle MRI lesions favored a diagnosis of MOGAD over MS and AQP4+ NMOSD. They further showed that diffuse medulla, pons, or midbrain MRI lesions occasionally occurred in MOGAD and AQP4-IgG-NMOSD but never in MS. Although these findings have revealed image-dependent differentiation, few studies have been conducted using conventional MRI and its possible integration with the deep learning technique into the clinical workflow.

Recent advances in artificial intelligence have prompted the development of deep learning-based algorithms designed for the automatic classification of demyelinating diseases based on conventional MRI (11, 18–20). For example, Kim et al. (18) constructed a three-dimensional convolutional neural network (CNN) deep-learning-based model using brain MRI and clinical information to differentiate NMOSD from MS, achieving a moderate accuracy of 71.1%, sensitivity of 87.8%, and specificity of 61.6%. Rocca et al. (20) also applied a deep-learning algorithm based on CNN using brain MRI to discriminate between MS and its mimics, including NMOSD, revealing the highest accuracy (98.8%) and specificity (98.4%), and the lowest false positive rate (4.4%) for MS.

Notably, these aforementioned studies mostly used brain MRI with or without incorporation of clinical information for image-based classification using a traditional CNN. There has been minimal exploration of integrated MRI sequences and multi-site consideration of neuroimaging protocols. To address this gap, we here proposed a novel deep-learning algorithm, according to Co-scale conv-attentional image Transformers (CoaT)-based network (21), which was trained on multi-sequence (coronal T2-weighted and sagittal T2-FLAIR) and multi-location (brain, cervicothoracic and thoracolumbar spinal cord) MRI. The combined image sequences represent a better reflection of a realistic clinical setting and may contribute to increased classification accuracy.

## 2 Materials and Methods

### 2.1 Ethics

This study was approved by the Ethics Committee of the Second Xiangya Hospital of Central South University, and the requirement for written informed consent was waived due to the retrospective nature of the study.

### 2.2 Participants

MR images and clinical data of patients with a CNS inflammatory demyelinating disease treated at the neurological department of our hospital between August 2013 and October 2021 were retrospectively reviewed for inclusion. The inclusion criteria were as follows: (a) confirmed diagnosis of MS, AQP4+ NMOSD, or MOGAD according to the 2017 McDonald diagnostic criteria (9), 2015 NMOSD criteria (10), and 2018 MOGAD diagnostic criteria (22), respectively; (b) at least one clinical demyelinating episode of the CNS (myelitis, optic neuritis, or encephalopathy); (c) AQP4 antibody and MOG antibody were tested using a cell-based assay method; and (d) all participants underwent MRI scanning of the brain and/or spinal cord. The exclusion criteria were: (a) both AQP4 and MOG antibody positivity; (b) incomplete clinical assessment; (c) a history of other neurological diseases, including stroke, epilepsy, traumatic brain injury, or psychiatric problems and (d) excessive artifacts in MR images.

Notably, images acquired during acute presentation of first attack or relapses were selected for inclusion in the analysis, whereas patients in their remission phase were not included. Clinical information on sex, age, Expanded Disability Status Scale (EDSS) score, onset times, and disease duration (calculated from the first symptom onset to the scan date) were also recorded.

### 2.3 MRI Acquisition

All brain and/or spinal cord imaging was sequences were performed on 1.5T (Magnetom Avanto, Siemens Healthcare, Erlangen, Germany; uMR 588, Shanghai United Imaging Healthcare, Shanghai, China; GE Sigma Twin speed, GE Healthcare, Milwaukee, WI, USA) or 3.0T (Magnetom Skyra, Siemens Healthcare, Erlangen, Germany; Philips Achieva 3.0T X-Series, Phillips Healthcare, the Netherlands; uMR 790, Shanghai United Imaging Healthcare, Shanghai, China) MRI scanners in the Second Xiangya Hospital of Central South University. The MRI data included brain imaging with axial T2-weighted and coronal T2-FLAIR sequences, and spinal cord imaging with sagittal T2-weighted sequences. It is worth mentioning that when patients suspected with demyelinating diseases, a standard scanning protocol of routine brain and spinal MRI were always performed in our hospital regardless of the presence of neurological deficits. However, a small proportion of patients who met the aforementioned inclusion criteria were found with only brain or spinal cord MRI and also included in this analysis. This may be attributed to the fact that these patients only performed MRI scans of a single location according to the presence of relevant neurological deficits. There have been some variations in the acquisition parameter over the years. Detailed parameters of the brain and spinal cord MRI sequences were shown in **Table S1 in the Supplemental Material**.

### 2.4 Reference Standard and Image Interpretations

Two neuroradiologists (CXH and FT, with 4 and 6 years of working experience, respectively) and a neurologist (WL) with 28 years of working experience were involved in visual assessment of the brain lesions and differential classification of MS, AQP4+ NMSDO, and MOGAD patients. Images were reviewed using RadiAnt Dicom Viewer software (Version 2021.2, Medixant, Poland).

The assessment was based on T1WI, T2WI and T2-FLAIR MRI sequences of the brain, spinal cord, and optic nerve, along with clinical data (e.g., age, sex, disease duration, EDSS score, and laboratory testing results). In the diagnosis of clinically confirmed demyelinating disease, each specialist reviewed all relevant data in detail and made diagnostic decisions in accordance with the 2017 McDonald diagnostic criteria (9), 2015 NMOSD criteria (10), and 2018 MOGAD diagnostic criteria (22), respectively; in the case of any discrepancy, the data were jointly reviewed until an agreement was reached. The diagnosis based on these medical records was considered as the reference standard of this research.

### 2.5 Deep-Learning Model

Given most patients suspected with autoimmune demyelinating diseases had undergone MRI scans of both brain and spinal cord, the goal of the study is to develop a model that can handle the combination of brain and spinal cord MRI data for diagnosis, which is closer to the current clinical scenario. In the current analysis, we have developed the data pool consist of multimodal MRI data, which included brain T2WI, brain T2-FLAIR, cervicothoracic T2WI, and thoracolumbar T2WI; each patient had one or more types of these sequences. Moreover, MRI imaging manifestations of the lesions showed broad heterogeneity in terms of size, distribution and locations. To address this challenge, we used the state-of-art transformer network as the basic structure to build our multimodal model combined with weak-label multiple instance learning (MIL) strategy. This novel deep-learning model were designed to be versatile in handling with images of multiple sequences and scanning locations for differentiating among MS, NMOSD, and MOGAD from conventional MRI data.

#### 2.5.1 Data Preprocessing

The dataset was randomly split into a development set and a testing set at a ratio of 4:1. We first applied intensity normalization by z-score transformation within the non-zero region of the MR images. The normalized intensities of all voxels were set to have a mean of 0 and standard deviation (SD) of 1 for each MRI sequence. We then adjusted the intensity of these voxels and other outliers by clipping them to the range [1^st^ percentile of the image to 99^th^ percentile of the image]. We then performed background removal, where all voxels from the background regions outside of the non-zero region were set to –9 to ensure a uniform background intensity.

#### 2.5.2 Multiple Instance Learning Strategy

To address the challenge of weakly labeled data (i.e., patient-level prediction without lesion/region-level annotation), we introduced a former-established MIL strategy (23). MIL is a typical weakly supervised learning paradigm that was proposed to tackle the problem of abnormalities in various locations to complement the diagnosis of tuberculosis or chronic obstructive pulmonary disease (24, 25). Specifically, MIL used slice as the model input, which leads to increased size of data in the training stage. Together with the slice-level data augmentation, such as cropping, rotation, flip, lightness and other data enhancements, we were able to mitigate the issue of imbalanced samples between brain and spinal cord MRI scans. Therefore, the bag-level MixUp on the data had the same amount of training samples in each category (26, 27). Detailed illustration of MIL-CoaT Transformer Framework was shown in **Figure S1 in the Supplemental Material**.

#### 2.5.3 CoaT-Based Transformer Network

Transformer-based algorithms are state-of-the-art deep-learning algorithms for image recognition, including MRI (28, 29). In this study, the Siamese CoaT-based transformer network (21) was adopted as the basic network for feature extraction. By using shared parameters, the CoaT network can extract features from all instances in the same “bag,” and the attention pooling block is used to fuse extracted class tokens of all instances. Finally, the fully connected layer and softmax activation function were applied to obtain the bag-level prediction probability of the three categories. The detailed network architecture and descriptions can be found **Figures S2 and S3 in the Supplemental Material**.

#### 2.5.4 Implementation

To train the proposed MIL-CoaT model, we used Adam optimization with a batch size of 16 and learning rate of $5\times 10^{-4}\times \frac{global\;batch\;size}{512}$ . In the training phase, the Siamese CoaT-based network was initialized using the pre-trained parameters of ImageNet (30), and cross-entropy loss was used. To address the problem of sample imbalance, the number of samples in each category was guaranteed to be consistent in each training iteration cycle. Therefore, the samples of the categories with smaller sample size were transformed and reused through data augmentation technique which can be treated as the new samples in iterative cycles. To test the deep learning model, we selected the middle slice from each sub-part as an instance in the MIL setting to construct the input sample. During the training and testing process, our deep-learning model was implemented using the popular open-source PyTorch framework and was run on four Nvidia GTX 1080Ti GPUs. The code of the model in this study have been uploaded to GitHub and are available at https://github.com/TXVision/Demyelinating_Diseases_Classification_MRI.

### 2.6 Reader Experiment

To assess the performance of our proposed deep learning model in the classification of demyelinating diseases, we recruited two neuroradiologist SNC and HYL (with 7 and 13 years of working experience, respectively) in the reader experiment. Briefly, the raters who were blinded to the patients’ clinical status independently reviewed all cases in the test dataset and were asked to classify subtypes of the demyelinating diseases with an assigned confidence score (0%-100%) for each class. The sum of the scores should equal to 100% (e.g., MS: 70%; NMOSD: 20%, MOGAD: 10%).

### 2.7 Statistical Analysis

Statistical analyses were performed using SPSS software (version 26.0; SPSS Inc., Chicago, IL, USA). Descriptive statistics are presented as frequencies and percentages for categorical variables and as means and SD for continuous variables. Differences in categorical variables between groups were analyzed using the Pearson chi-square test or Fisher’s exact test, as appropriate. Differences in continuous variables were analyzed using the Mann-Whitney U test. The diagnostic performance of the proposed model was assessed using the receiver operating curve (AUC) with the 95% confidence interval (CI). The optimal cut-off value was chosen using the Youden index (sensitivity + specificity -1), as previously in Huang et al. (31). Thus, sensitivity, specificity; accuracy, positive predictive value (PPV), negative predictive value (NPV), and F1 score were calculated accordingly.

For performance evaluation in multi-category classification of the raters, the category with the highest probability value among the rater’s output was regarded as the differential diagnosis of the disease. Thereafter, confusion matrices were drawn and overall accuracy were compared between the fusion model and the raters using the McNemar test, with p-value < 0.05 indicating the statistically significant difference. Confusion matrix deriving Matthew’s correlation coefficient (MCC) and Cohen’s kappa coefficient (Kappa) were also recorded and compared.

## 3 Results

### 3.1 Demographic and Clinical Characteristics

**Figure 1:**
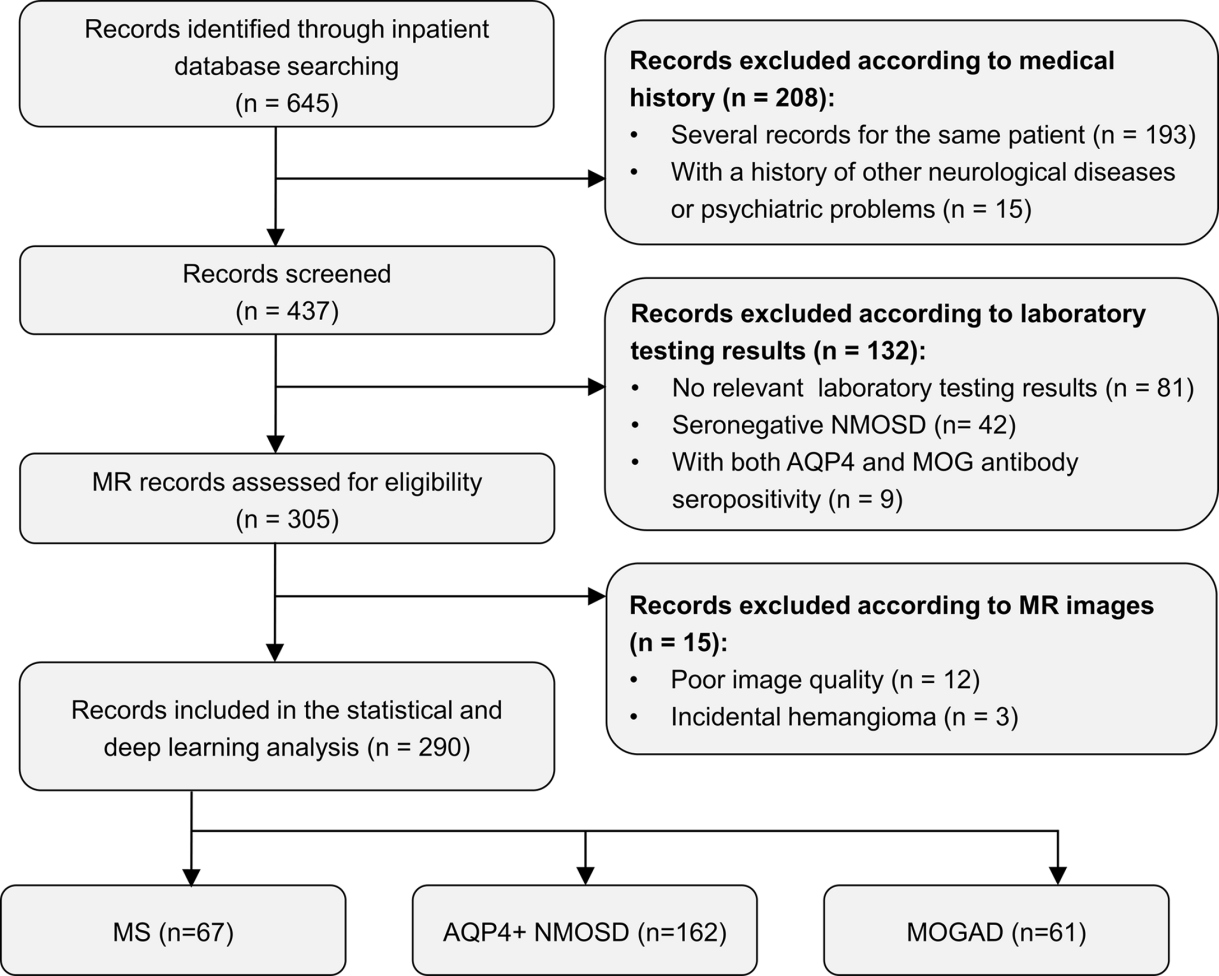
Flow chart of the selection process of included participants. AQP4+ NMOSD, aquaporin 4 positive neuromyelitis optica spectrum disorders; MOGAD, myelin oligodendrocyte glycoprotein antibody associated disease; MR, magnetic resonance; MS, multiple sclerosis.

A total of 290 patients with CNS inflammatory demyelinating diseases, including 67 with MS, 162 with AQP4+ NMOSD, and 61 with MOGAD, were included for analysis. **Figure 1** shows a flowchart of the selection process of the included patients. All patients were randomly assigned to the development set (composed of training and validation sets), including 231 patients (53 with MS, 129 with AQP4+ NMOSD, and 49 with MOGAD) and the testing set, including 59 patients (14 with MS, 33 with AQP4+ NMOSD, and 12 with MOGAD) at a ratio of 4:1.

The demographic and clinical characteristics of all patients are summarized in **Table 1**. There were no significant differences in age, sex, disease duration, onset times, EDSS score, and the presence of visual disturbance for the MS, AQP4+ NMOSD, and MOGAD groups, respectively, between the development and testing datasets.

**Table 1:** Demographic and clinical characteristics in patients with MS, AQP4+ NMOSD, and MOGAD.

	Development Set (n = 231)			Testing Set (n = 59)			
	MS	AQP4+ NMOSD	MOGAD	MS	AQP4+ NMOSD	MOGAD	p value^*^
**Clinical characteristics**							
No. of patients, n	53	129	49	14	33	12	–
Age, mean ± SD, years	33.11 ± 12.83	44.21 ± 14.10	23.31 ± 18.09	34.50 ± 14.03	42.12 ± 15.30	27.33 ± 15.74	> 0.05
Adults (≥18 years), n (%)	50 (94.34%)	126 (97.67%)	22 (44.90%)	14 (100%)	31 (93.94%)	7 (58.33%)	–
Sex (male/female)	28/25	10/119	20/29	8/6	3/30	6/6	> 0.05
Disease duration, mean ± SD, months	31.74 ± 50.41	26.76 ± 51.80	14.15 ± 37.74	46.96 ± 48.99	38.50 ± 79.02	10.33 ± 24.11	> 0.05
Onset times, mean ± SD	1.96 ± 0.88	1.90 ± 1.34	1.47 ± 0.92	2.14 ± 0.66	1.73 ± 1.21	1.17 ± 0.39	> 0.05
First attack, n (%)	18 (33.96%)	66 (51.16%)	36 (73.47%)	2 (14.29%)	20 (60.61%)	10 (83.33%)	–
Second attack, n (%)	22 (41.51%)	35 (27.13%)	6 (12.24%)	8 (57.14%)	7 (21.21%)	2 (16.67%)	–
≥3 attacks, n (%)	13 (24.53%)	28 (21.71%)	7 (14.29%)	4 (28.57%)	6 (18.18%)	0 (0)	–
EDSS score at the time of MRI, mean ± SD	3.53 ± 1.87	5.70 ± 2.22	2.45 ± 1.28	4.14 ± 1.62	4.86 ± 1.99	2.54 ± 1.66	> 0.05
Visual disturbance, n (%)	21 (39.62%)	48 (37.21%)	27 (55.10%)	4 (28.57%)	9 (27.27%)	5 (41.67%)	> 0.05
**MRI scanning information**							
No. of MRI sequences	178	411	166	45	112	41	–
Brain + spinal cord, n (%)	39 (73.58%)	91 (70.54%)	36 (73.47%)	10 (71.43%)	26 (78.79%)	9 (75.00%)	–
Brain only, n (%)	13 (24.53%)	6 (4.65%)	12 (24.49%)	4 (28.57%)	1 (3.03%)	3 (25.00%)	–
Cervicothoracic and/or thoracolumbar spinal cord only, n (%)	1 (1.89%)	32 (24.81%)	1 (2.04%)	0 (0)	6 (18.18%)	0 (0)	–
MR scanner field strength							
3.0 T scanners	12	50	28	2	13	3	–
1.5 T scanners	41	79	21	12	20	9	–

*Significant difference (p < 0.05) of each clinical variable in the MS, AQP4+ NMOSD, and MOGAD groups, respectively, between the development and testing datasets.

AQP4+ NMOSD, aquaporin 4 positive neuromyelitis optica spectrum disorders; EDSS, expanded disability status scale; MOGAD, myelin oligodendrocyte glycoprotein antibody associated disease; MRI, magnetic resonance imaging; MS, multiple sclerosis; SD, standard deviation.

All patients underwent MRI scanning of the brain and/or spinal cord, and a total of 953 sequences were analyzed. Among the 290 patients included in the study, a total of 211 patients (72.8%) who have undergone both brain and spinal cord MRI scans. Among 250 (86.2%) patients with brain MRI and 251 (86.6%) patients with spinal cord MRI, there were 188/250 (75.2%) patients and 214/251 (85.3%) patients containing abnormal lesions, respectively. MR images of 14 (4.8%) patients from the AQP4+ NMOSD or MOGAD group showed no visible lesions in both the brain and spinal cord.

### 3.2 Diagnostic Performance of the MIL-Transformer Network Using Single- or Multi- Site MRI

We chose the Youden index as the optimal cut-off value to retrieve the variety of measurements including accuracy, sensitivity, specificity, PPV, NPV, and F1 score, which was shown in **Table 2**.

**Table 2:** Diagnostic performance of our proposed MIL-CoaT transformer model based on different inputs in classification of MS, AQP4+ NMOSD and MOGAD.

One-vs.-rest classification	ROC_AUC (95% CI)	Accuracy (%)	Sensitivity (%)	Specificity (%)	PPV (%)	NPV (%)	F1
**Brain MRI as model inputs**							
MS vs. others	0.936 (0.855, 0.990)	88.9	78.6	92.5	78.6	92.5	0.786
AQP4+ NMOSD vs. others	0.940 (0.870, 0.986)	87.0	78.6	96.2	95.7	80.6	0.863
MOGAD vs. others	0.782 (0.606, 0.938)	85.2	58.3	92.9	70.0	88.6	0.636
**Spinal cord MRI as model inputs**							
MS vs. others	0.724 (0.539, 0.897)	74.5	70.0	75.6	41.2	91.2	0.519
AQP4+ NMOSD vs. others	0.689 (0.520, 0.833)	70.6	71.9	68.4	79.3	59.1	0.780
MOGAD vs. others	0.714 (0.494, 0.919)	82.4	55.6	88.1	50.0	90.2	0.526
**Combined brain and spinal cord MRI as model inputs**							
MS vs. others	0.933 (0.848, 0.991)	84.7	92.9	82.2	61.9	97.4	0.743
AQP4+ NMOSD vs. others	0.942 (0.879, 0.987)	88.1	87.9	88.5	90.6	85.2	0.892
MOGAD vs. others	0.803 (0.629, 0.949)	72.9	83.3	70.2	41.7	94.3	0.556

AQP4+ NMOSD, aquaporin 4 positive neuromyelitis optica spectrum disorders; AUC, area under curve; CI, confidence interval; MOGAD, myelin oligodendrocyte glycoprotein antibody associated disease; MS, multiple sclerosis; ROC, receiver operating characteristic curve.

**Figure 2:**
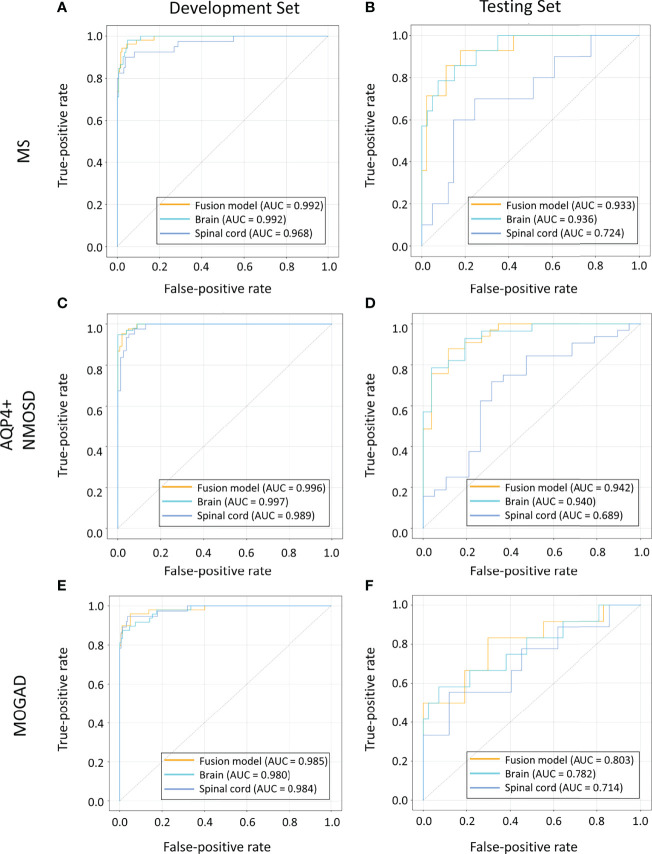
ROC curves of the models based on brain, spinal cord, and combined MRI sequences in the cohorts of patients with MS **(A, B)**, AQP4+ NMOSD **(C, D)** and MOGAD **(E, F)**. AQP4+ NMOSD, aquaporin 4 positive neuromyelitis optica spectrum disorders; MOGAD, myelin oligodendrocyte glycoprotein antibody associated disease; MRI, magnetic resonance imaging; MS, multiple sclerosis; ROC, receiver operating characteristic.

For AQP4+ NMOSD, the ROC curves (**Figure 2**) showed that deep-learning models trained with individual brain and spinal cord MRI had AUCs of 0.940 (95%CI: 0.870, 0.986) and 0.689 (95%CI: 0.520, 0.833) respectively. In comparison, the deep-learning fusion model provided better diagnostic performance with AUC of 0.942 (95%CI: 0.879, 0.987) for AQP4+ NMOSD.

When identifying MOGAD, the AUCs of the models trained with individual brain and spinal cord MRI were 0.782 (95% CI: 0.606, 0.938) and 0.714 (95% CI: 0.494, 0.919), respectively. In comparison, the deep-learning fusion model exhibited superior diagnostic performance with AUC of 0.803 (95%CI: 0.629, 0.949) for MOGAD.

The deep-learning model based on the spinal cord MRI had AUC of 0.724 (95%CI: 0.539, 0.897) for MS, which was small than that of the model trained with the brain MRI with AUC of 0.936 (95%CI: 0.855, 0.990) and the combined sequences (the fusion model) with AUC of 0.933 (95%CI: 0.848, 0.991). The AUC of the fusion model was marginally smaller than that of the model trained with brain MRI for MS.

### 3.3 Multi-Category Classification Comparison of the Deep-Learning Model Against Neuroradiologists

Multi-category classification using the proposed MIL-transformer network also exhibited better performance in the fusion model. Among the models trained with MRI on individual and combined locations, the fusion model exhibited better performance with an accuracy of 81.4% (Kappa 0.666, MCC 0.682). On the contrary, the model using individual brain or spinal cord MRI as input had an accuracy of 75.9% (Kappa 0.605, MCC 0.623) and 62.7% (Kappa 0.202, MCC 0.215), respectively.

**Figure 3:**
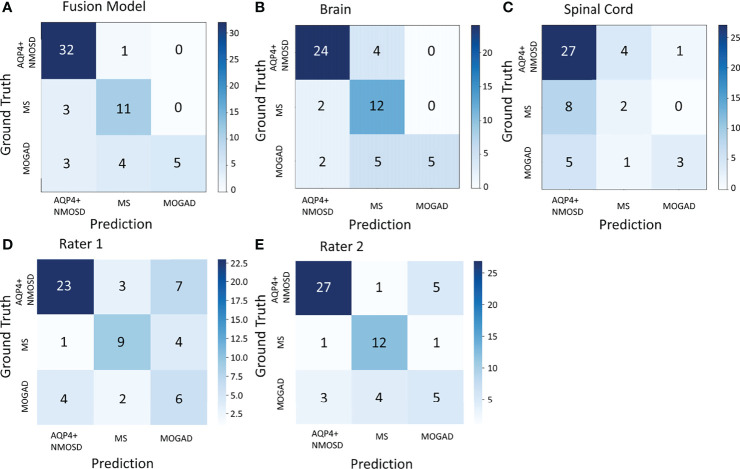
The confusion matrix of the fusion model in the test dataset of 59 patients **(A)**, the model based on the brain MRI **(B)**, the model based on the spinal cord MRI **(C)**, and human rater 1 and 2 **(D, E)**. AQP4+ NMOSD, aquaporin 4 positive neuromyelitis optica spectrum disorders; MOGAD, myelin oligodendrocyte glycoprotein antibody associated disease; MS, multiple sclerosis.

We also compared the classification performance obtained with the proposed models versus that of human raters. In the same test dataset of 59 patients, the overall accuracy of the deep-learning model was higher than that of rater 1 and comparable to rater 2 (81.4% vs. 64.4% for rater 1, p = 0.04 < 0.05 and 81.4% vs. 74.6% for rater 2, p = 0.388 > 0.05). Meanwhile, as shown in **Figure 3**, the confusion matrix of the fusion model exhibited a higher Kappa of 0.666, MCC of 0.682 than that of human raters, who had a Kappa of 0.426, MCC of 0.431 for rater1 and Kappa of 0.576, MCC of 0.578 for rater 2.

### 3.4 Visual Explanation of the Deep-Learning Model

**Figure 4:**
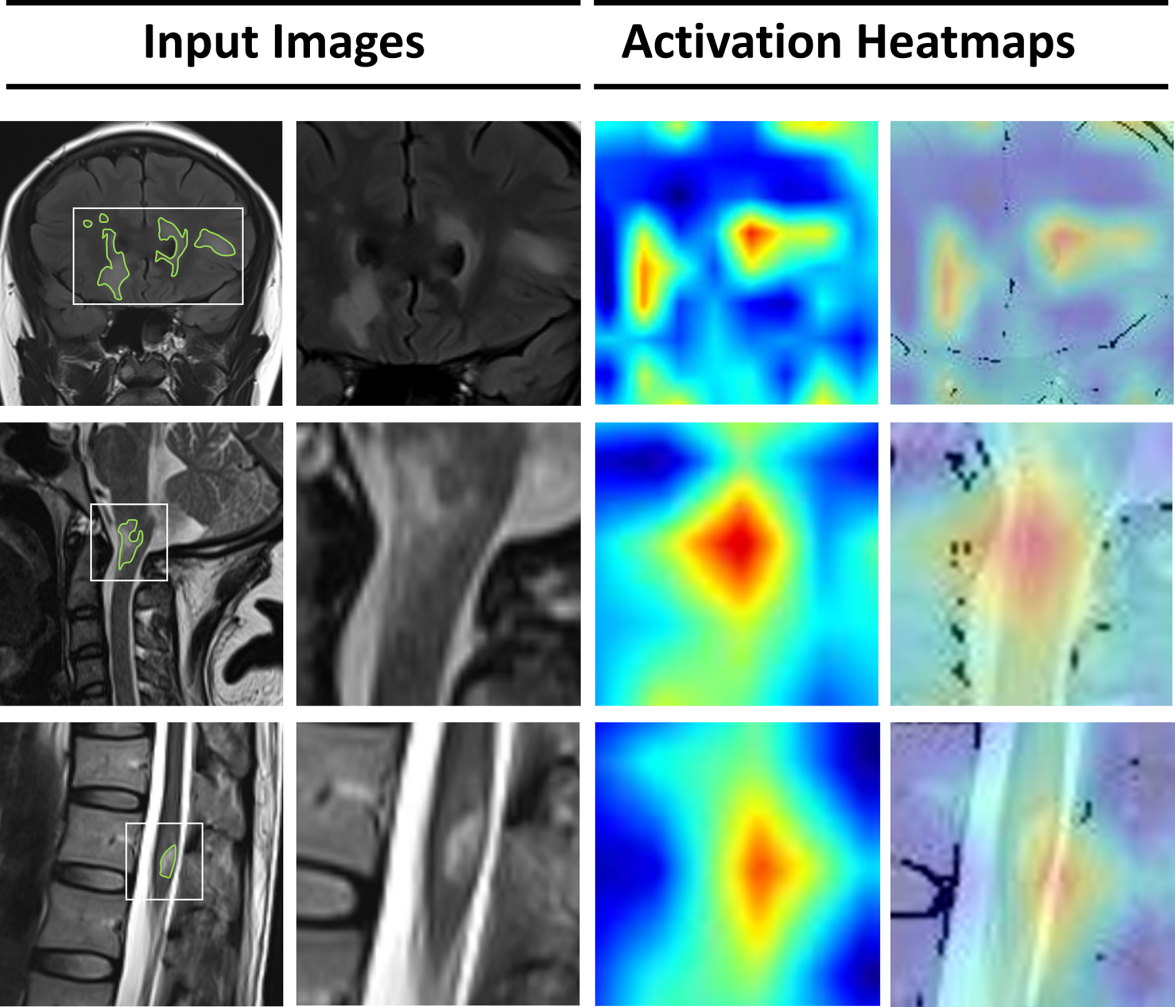
Visualization of features extracted by the deep-learning model from the input images. From the left, the first column represents the original MRI slices with manual annotations of lesions in the brain, cervical spinal cord, and thoracic spinal cord. In the second column, a smaller patch is cropped around the lesions. The third column represents the activation heatmaps. The color depth of the heatmaps represents the possibility of predicted lesions by the model. The fourth column overlaps the activation mapping with the original MRI for better visual reference.

The lack of transparency in deep learning can be overcome by applying gradient-weighted class activation (Grad-CAM) to visualize feature extraction using an activation heatmap (32). As shown in **Figure 4**, lesions in the brain and spinal cord manifested as relatively dark color in the Grad-CAM results. Insights generated from Grad-CAM were compared with manual annotations, and the results indicated that the model focuses on these lesions when distinguishing demyelinating diseases. This can help us to gain an understanding of the regions within the MR images that are responsible for network predictions.

## 4 Discussion

In this study, we proposed transformer-based deep-learning model to differentiate among MS, AQP4+ NMOSD and MOGAD based on conventional brain and spinal cord MRI. This novel transformer-based model architecture was designed to be versatile in handling with images of multiple sequences and scanning locations whichever were available at clinical practice, for differentiating among MS, NMOSD, and MOGAD at a high accuracy. The fusion model using images of combined locations exhibited significantly higher accuracy than the models trained with a single location MRI as well as two experienced radiologists, which referred to possible alternative tool in assisting clinical decisions for a fast and accurate treatment referral.

To our knowledge, this is the first study to use an ensemble-location approach in the task of multi-category classification to improve the differential diagnosis of CNS inflammatory demyelinating diseases. Given patients suspected with demyelinating disease may undergo brain and/or spinal cord MRI scans, the model was developed to handle with the data in the single- or multi- location manner on conventional MR images. When MR images at one location were taken, our results showed that taking brain images as model inputs had a relative higher accuracy in classifying these three conditions than using spinal cord images only (pooled accuracy: 75.9% vs. 62.7%). Moreover, when multi-location MR images were available, the fusion model demonstrated an improved accuracy of 81.4%, which was comparable compared to the performance of two neuroradiologists (accuracy: 64.4% for rater 1 and 74.6% for rater 2). Meanwhile, the fusion model exhibited higher Kappa and MCC than that of human raters.

Previous studies have used only brain or spinal cord images (11, 20), along with accompanying clinical variables (18, 33) for prediction. Among these studies, Rocca et al. (20) used CNN and achieved an accuracy of 98.8% for the differential diagnosis of MS from NMOSD. However, it shall be noted that this high diagnostic performance of these models may be attributed to obvious differences in lesion volume and distribution, which may lead to over-estimated classification accuracy of the models. This potential bias was addressed in our study by enrolling images from patients whose MRI were taken at multi-location and the features extracted broad heterogeneity in lesion distribution were regarded as shared manifestations among target demyelinated disease.

Deep-learning algorithms are an active area of research in medical image processing. These algorithms can extract information from conventional MR images, including features that cannot be recognized by the human eye, and help to make a more accurate diagnosis (34), prognosis evaluation (35) and therapeutic guidance (36). Our proposed multimodal MIL-CoaT deep-learning network can perform multicategory classification using hybrid MRI sequences. This model was developed based on an MIL strategy and incorporated the CoaT transformer as the basic network structure. Specifically, the MIL strategy used slice as the model input, which leads to increased size of data in the training stage. Together with the slice-level data augmentation, we were able to mitigate the issue of imbalanced samples between brain and spinal cord MRI scans. Furthermore, during the training stage, each epoch has the same amount of training sample at each group. The samples of the categories with smaller sample size were transformed and reused through data augmentation technique which can be treated as the new samples in iterative cycles. Moreover, the CoaT-based transformer structure has the characteristics of dynamic attention and global context fusion, which are not available with a traditional CNN. Thus, by combining the extracted features and subsequent instance-wise feature fusion using attention pooling, we obtained a model with improved generalization ability.

We reviewed misclassified cases to determine their imaging characteristics and speculate possible reasons. We found that the cases misclassified as AQP4+ NMOSD can exhibit certain characteristics such as the presence of brain lesions adjacent to the fourth ventricle, and multiple short segment lesions that fused into long segment lesions in the spinal cord, which may resemble the appearance of AQP4+ NMOSD. Moreover, the presence of severe brain atrophy, and involvement of cortical or juxtacortical regions may be a possible cause of being misdiagnosed as MS.

This study indeed had some limitations. First, optic nerve lesions may exist in CNS demyelinating diseases, whereas the limited imaging data of optic nerve MRI were not suitable for inclusion in big data analysis. Second, the lack of thin-section MRI data due to time-cost issues may have resulted in lower image resolution and less precise information. However, it shall be mentioned that in most hospitals in China, the conventional MRI protocol with thick slices was utilized for the diagnosis of patients suspected with demyelinated diseases. This is attributed to larger number of patients and longer waiting times compared to western countries. Under this condition, we focused on research-quality MR images and developed the deep learning model that could be used to classify major types of demyelinated disease with high accuracy. Third, the loss of accuracy with the fusion model including spinal cord MRI in MS cases may have been attributed to the limited sample size and the fact that some MS patients did not have lesions in the spinal cord. This would be an important feature for co-learning to ensure reliability in applying the model to clinical practice.

## 5 Conclusion

Overall, our results provide evidence that deep-learning networks may be used for differential diagnosis based on brain and spinal cord MRI for patients with MS, AQP4+ NMOSD, and MOGAD. To our knowledge, this is the first study to apply the newly proposed MIL-CoaT transformer-based deep-learning algorithm to conventional MRI of multiple locations and sequences in attempt to solve the clinical challenge of diagnosing CNS demyelinating diseases. This evidence is also expected to motivate future research for delving into the clinical and radiological basis of deep-learning networks, as well as to validate the findings with a prospective study design.

## Data Availability Statement

The original contributions presented in the study are included in the article/**Supplementary Material**. Further inquiries can be directed to the corresponding authors.

## Ethics Statement

The studies involving human participants were reviewed and approved by the Ethics Committee of the Second Xiangya Hospital of Central South University. Written informed consent from the participants’ legal guardian/next of kin was not required to participate in this study in accordance with the national legislation and the institutional requirements.

## Author Contributions

JL and WL conceived and designed the study. XC collected imaging and clinical data. CH, FT, and WL reviewed the data. BL, WC, and RY analyzed and interpreted the data. CH drafted the manuscript. All authors contributed to the article and approved the final version.

## Funding

This work was supported by the Clinical Research Center for Medical Imaging in Hunan Province (2020SK4001). Leading talents of scientific and technological innovation in Hunan Province in 2021 (2021RC4016). The accurate localization study of mild traumatic brain injury based on deep learning through multimodal image and neural network (2021gfcx05).

## Conflict of Interest

Authors BL, WC, and RY were employed by Infervision Medical Technology Co., Ltd.

The remaining authors declare that the research was conducted in the absence of any commercial or financial relationships that could be construed as a potential conflict of interest.

## Publisher’s Note

All claims expressed in this article are solely those of the authors and do not necessarily represent those of their affiliated organizations, or those of the publisher, the editors and the reviewers. Any product that may be evaluated in this article, or claim that may be made by its manufacturer, is not guaranteed or endorsed by the publisher.
