# Supplementary material for: Transformer-Based Deep-Learning Algorithm for Discriminating Demyelinating Diseases of the Central Nervous System With Neuroimaging
Source: Front Immunol. 2022 Jun 14;13:897959. doi: 10.3389/fimmu.2022.897959 (PMC9238435; doi:10.3389/fimmu.2022.897959)
Supplement: Supplementary file 1 [file DataSheet_1.pdf]

Supplemental Material

Model architecture

As shown in Figure S2, the Siamese CoaT-based Network uses CoaT (1) as the basic network for feature extraction, which extracts features from all instances in the same "bag" by sharing parameters. Thus, it uses the Attention Pooling model to extract the class tokens of all instances, followed by feature fusion and the fully connected layer along with the softmax activation function to obtain the bag-level three-category prediction probability. Among them, the architecture of Serial Block is shown in Figure S3. Briefly, we firstly used the patch embedding layer to downsample the input feature maps, and then input the obtained tokenized features to multiple conv-attention and feed-forward layers for processing. Detailed training process was explained previously in Xu et al (1).

Reference:

(1) Xu W, Xu Y, Chang T, Tu Z. Co-Scale Conv-Attentional Image Transformers. ArXiv E-print (2021). doi: 10.48550/arXiv.2104.06399

False positive and negative analysis

In the test dataset, a total of 11 cases (18.6% of the 59 cases) had discordant findings between the deep learning model and the reference standard. Among them, 3 MS cases (21.4%) were misclassified as AQP4+ NMOSD; only one AQP4+ NMOSD case (3.0%) was misclassified as MS; In the category of MOGAD (12 cases), 3 cases (25.0%) were misclassified by the model as AQP4+ NMOSD and 4 cases (33.3%) were misclassified as MS. We have summarized imaging characteristics of the misclassified cases and speculated possible reasons. Firstly, brain lesions that exhibited certain characteristics (adjacent to the fourth ventricle, or multiple short segment lesions that fused into long segment lesions in the spinal cord) may resemble the similar appearance of AQP4+ NMOSD; secondly, the presence of severe brain atrophy, and the involvement of cortical or juxtacortical regions may be a possible cause of being misdiagnosed as MS.

Table S1 Acquisition parameters of different MRI sequences for patients analyzed in this study

Sequences	Repetition time (ms)	Echo time (ms)	Flip angle	Number of slices	Section thickness (mm)
Brain axial T2WI	2800–6287	80–131	90°–150°	13–28	3–7
Brain coronal T2-FLAIR	3700–9240	87–134	90°–150°	14–36	3–8
Cervicothoracic spinal cord sagittal T2WI	1700–8000	90–120	90–160°	8–26	3–5
Thoracolumbar spinal cord sagittal T2WI	1800–4500	82–125	90–160°	7–25	3–4

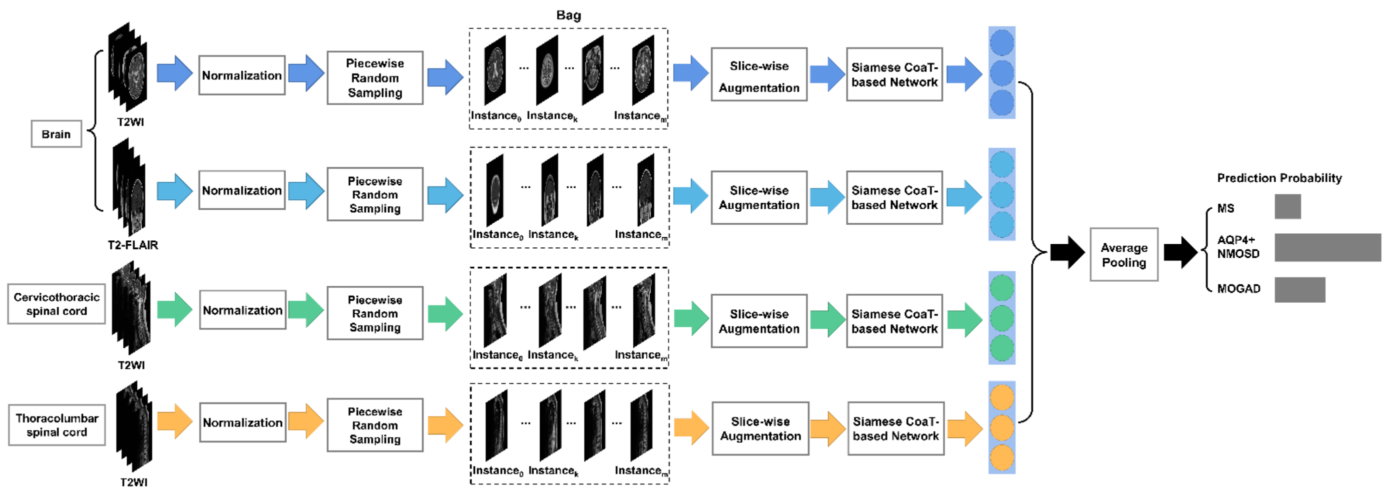

Figure S1 Illustration of our proposed MIL-CoaT Transformer Framework. For multi-instance learning, each MRI scan was divided into M sub-parts of an equal height, and one slice was randomly selected from each sub-part as one instance. Multi-instance learning assumes that labels for collections of instances (bags) are available, whereas labels for individual instances are missing. In our case, we assumed that each bag from a positive-class sample contained at least a few slices with positive class-specific information, whereas each bag from a negative-class sample did not contain any slices with positive class-specific information.

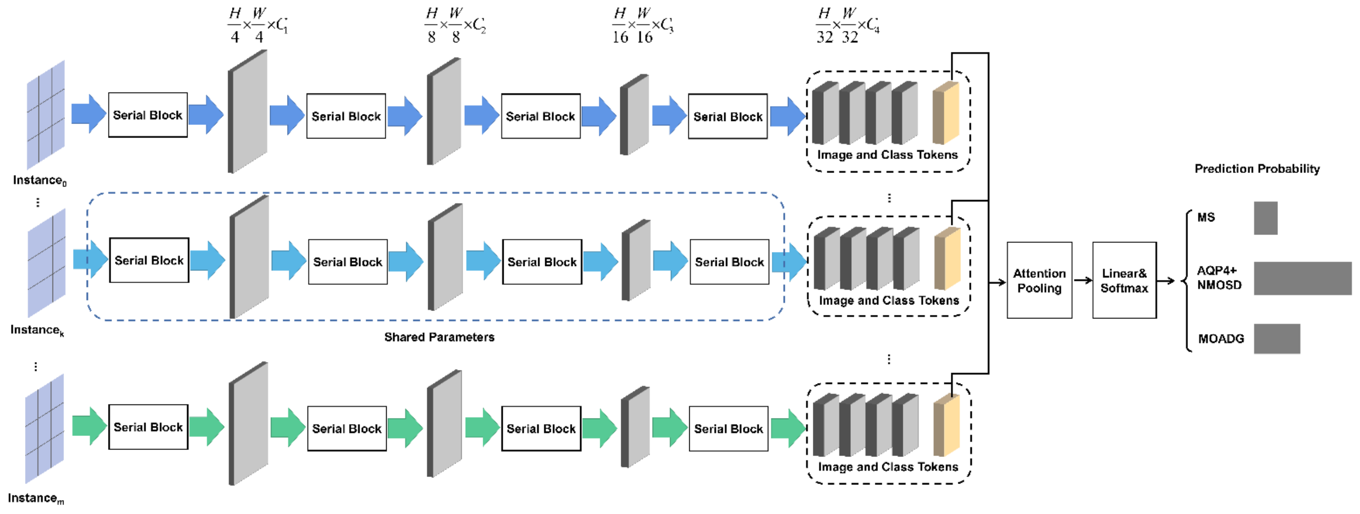

Figure S2 Illustration of Siamese CoaT-based Network

Siamese CoaT-based Network was implemented with the Attention-based MIL pooling (1), which was designed to find the "resonance" between instance-level feature vectors in a bag. It namely finds the most relevant embedding features for the three-class recognition. The attention-based MIL pooling computes the following function:

$$M = \tanh(H)$$

$$\alpha = softmax(w^T M)$$

$$r = H\alpha^T$$

where H denotes a matrix consisting of output vectors [h_1, h_2, ..., h_T]; T denotes the input sequence length; r denotes a weighted sum of these output vectors; w is a trained parameter vector and w^T is a transpose.

Reference:

(1) Ilse M, Tomczak JM, Welling M. Attention-Based Deep Multiple Instance Learning. Proceedings of the 35th International Conference on Machine Learning. (2018): PMLR 80:2127-36. doi: 10.48550/arXiv.1802.04712.

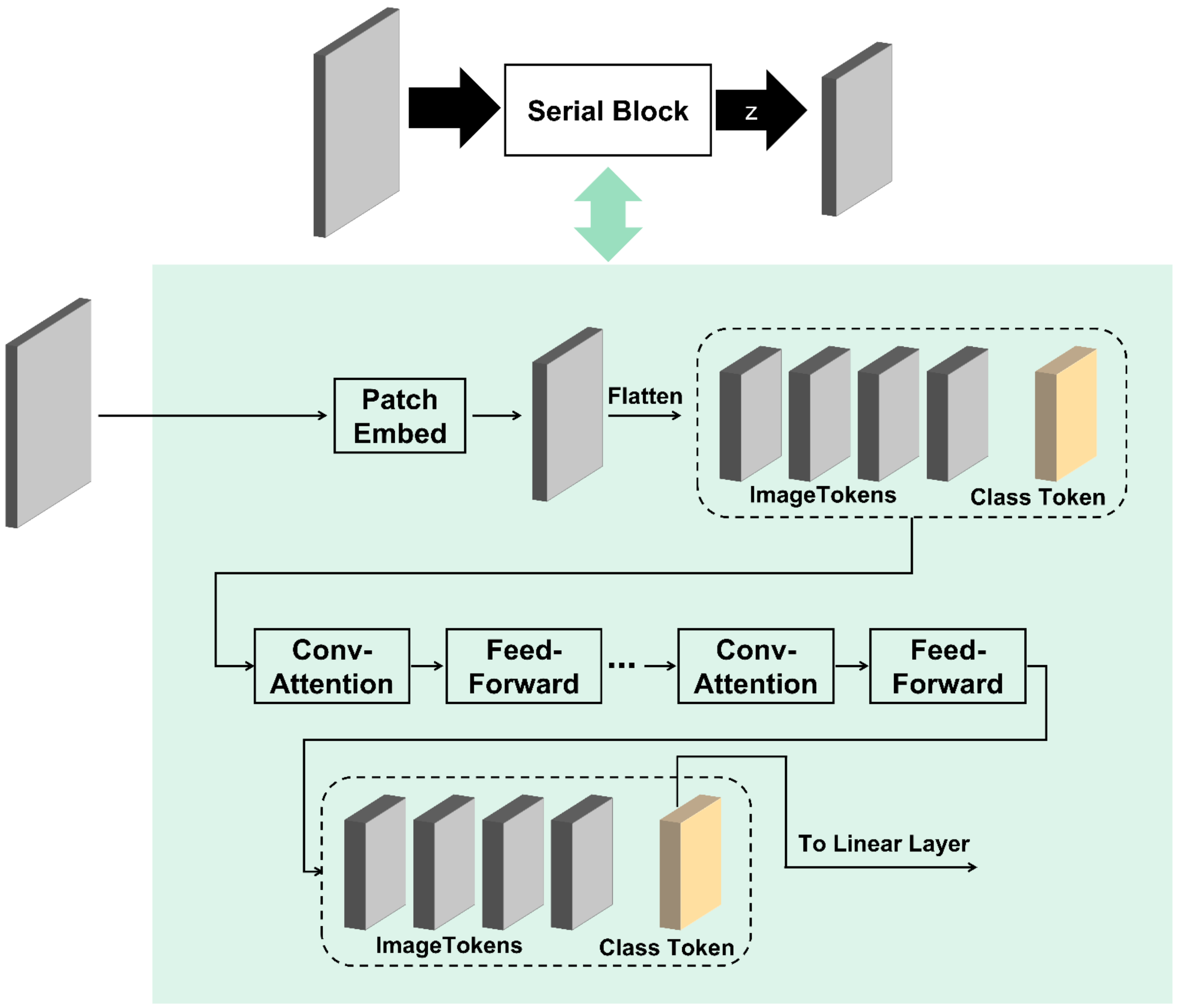

Figure S3 Illustration of Serial Block
